# Effects of Low Molecular Weight Peptides from Red Shrimp (*Solenocera crassicornis*) Head on Immune Response in Immunosuppressed Mice

**DOI:** 10.3390/ijms241210297

**Published:** 2023-06-18

**Authors:** Rui Zhao, Shuoqi Jiang, Yunping Tang, Guofang Ding

**Affiliations:** Zhejiang Provincial Engineering Technology Research Center of Marine Biomedical Products, School of Food and Pharmacy, Zhejiang Ocean University, Zhoushan 316022, China; zhaorui@zjou.edu.cn (R.Z.); jsq_sxty@163.com (S.J.); dinggf2007@163.com (G.D.)

**Keywords:** low molecular weight peptides, immunoenhancement, cyclophosphamide, *Solenocera crassicornis*, signal pathways

## Abstract

This study aimed to investigate the immunoenhancement effects of low molecular weight peptides (SCHPs-F1) from red shrimp (*Solenocera crassicornis*) head against cyclophosphamide (CTX)-induced immunosuppressed mice. ICR mice were intraperitoneally injected with 80 mg/kg CTX for 5 consecutive days to establish the immunosuppressive model and then intragastrically administered with SCHPs-F1 (100 mg/kg, 200 mg/kg, and 400 mg/kg) to investigate its improving effect on immunosuppressed mice and explore its potential mechanism using Western blot. SCHPs-F1 could effectively improve the spleen and thymus index, promoting serum cytokines and immunoglobulins production and upregulating the proliferative activity of splenic lymphocytes and peritoneal macrophages of the CTX-treated mice. Moreover, SCHPs-F1 could significantly promote the expression levels of related proteins in the NF-κB and MAPK pathways in the spleen tissues. Overall, the results suggested that SCHPs-F1 could effectively ameliorate the immune deficiency caused by CTX and had the potential to explore as an immunomodulator in functional foods or dietary supplements.

## 1. Introduction

The immune system resists the invasion of foreign pathogens through multiple defense lines composed of the innate and adaptive immune systems [1]. The stability of the immune system is closely related to the physiological function of the body. However, this homeostasis may be affected by a variety of unfavorable factors, including obesity, psychology, and hormones [2,3,4]. Immunomodulators are synthetic, biological, or natural molecules that modulate immune responses in immune diseases and restore immune homeostasis. Chemically synthesized immunomodulators such as levamisole and dacarbazine are currently unsuitable for long-lasting clinical usage on account of adverse effects and instability concerns [5,6]. Thus, there is an urgent need to develop more stable and safer new immunomodulators to reduce the adverse reactions of immunotherapy.

In recent years, with the advancement of the food industry, it has gradually become a reality to improve human health by improving dietary conditions and exerting the physiological regulation function of the food itself [7]. Food-derived bioactive peptides, as an important component of functional foods, are of great value in the regulation of physiological functions, including anti-oxidation [8], anti-hypertension [9], anti-cancer [10], anti-inflammatory [11], anti-bacterial [12], etc. Currently, researchers have a strong interest in the high-value utilization of waste, and many reports have confirmed the feasibility of obtaining bioactive peptides from marine organisms and their processing by-products [13,14]. Hou et al. [15] reported that three immunomodulatory peptides (NGMTY, NGLAP, and WT) isolated and purified from Alaska pollock frame hydrolysate had high lymphocyte proliferation activity. Kim et al. [16] reported that tuna cooking drip and its enzymatic hydrolysate not only increased the proliferation rate of mouse splenocytes but also promoted the levels of interleukin (IL)-2, IL-10, and immunoglobulin G (IgG). Yu et al. [17] reported that RVAPEEHPVEGRYLV, a *Cyclina sinensis* polypeptide, alleviates CTX-induced immunosuppression by enhancing humoral and cellular immunity in mice. Therefore, it has a favorable development potential to obtain immunomodulatory peptides by enzymatic hydrolysis of marine biological proteins.

In the South China Sea, *Solenocera crassicornis* is a valuable commercial shrimp species that is mainly processed into shrimp flesh [18]. However, underutilized shrimp heads and other processing by-products are discarded in large quantities, aggravating resource waste and environmental pollution. Song et al. [19] extracted astaxanthin from by-products such as the head of *S. crassicornis* with good antioxidant activity and effectively improved paracetamol-induced acute liver injury in rats. We prepared shrimp head peptide (SCHPs-F1) with antioxidant activity by enzymatic extraction, and SCHPs-F1 could alleviate CTX-induced liver and kidney damage [20,21]. However, the immunomodulatory effect of SCHPs-F1 in vivo has not been thoroughly demonstrated. In this study, we used CTX-induced immunocompromised mice to explore the in vivo immunoregulatory activity of SCHPs-F1 and to explore its potential regulatory mechanism through the expression of NF-κB and MAPK pathway-related proteins so as to offer a theoretical framework for future immunoregulatory functional food preparations employing shrimp by-products.

## 2. Results

### 2.1. Characterization of SCHPs-F1

**Figure 1:**
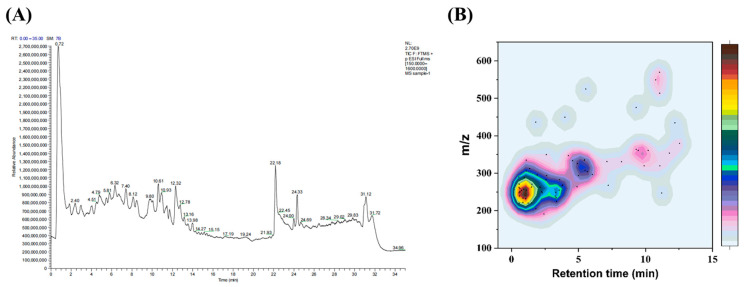
Total ion current diagram (**A**) and scattered point heat diagram (**B**) of SCHP-F1 by LC-MS/MS.

SCHPs-F1 (less than 1 kDa) was prepared according to our previous report using pepsin and trypsin, which consisted of peptides within the scope of 180–500 Da (72.5%) and 500–1000 Da (27.5%) [20]. Additionally, 16 kinds of amino acids are found in SCHPs-F1, among which the contents of essential amino acids and branched-chain amino acids are 28.94 g/100 g and 11.34 g/100 g, respectively [20]. Furthermore, UPLC-MS/MS was used to characterize the sequences of immunomodulatory peptides in SCHPs-F1 [22,23], which is equipped with a Q Exactive hybrid quadrupole-orbitrap mass spectrometer and a Dionex Ultimate 3000 UHPLC system (Thermo Fisher Scientific, Waltham, MA, USA), Figure 1A. Table S1 shows that 71 peptides are identified in this study, most of which are dipeptides and tripeptides, and the mass-to-charge ratios are concentrated in the range of 200–300 (Figure 1B).

### 2.2. Effect of SCHPs-F1 on Body Weight and Immune Organ Index

**Figure 2:**
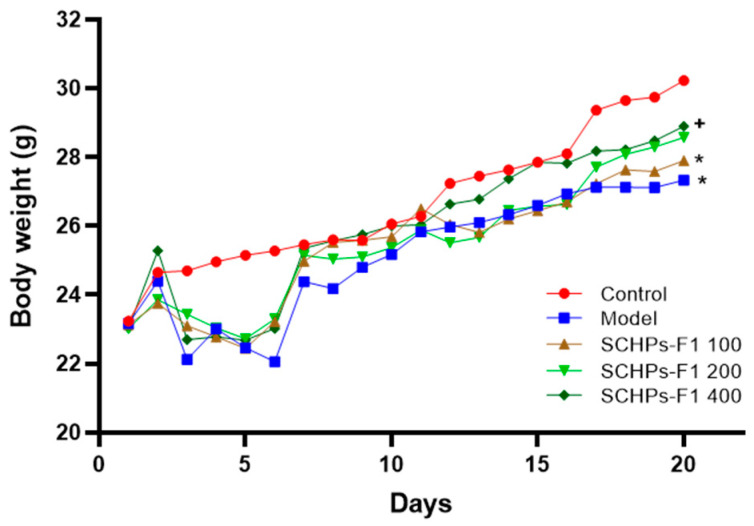
Changes in body weight of mice. All data are expressed as mean ± SD (*n* = 8 for each group, * *p* < 0.05 vs. control group, and ^+^ *p* < 0.05 vs. model group).

The body weight of the mice is substantially reduced following treatment with CTX (Figure 2) compared with the control group (26.81 ± 1.17 vs. 29.74 ± 1.48, *p* < 0.05). The final body weight of mice administered with 400 mg/kg SCHPs-F1 is significantly higher than the CTX group (28.46 ± 3.79 vs. 26.81 ± 1.17, *p* < 0.05), manifesting that SCHPs-F1 alleviated the immunological injury produced by CTX.

As illustrated in Table 1, CTX markedly inhibits the weight of the mouse spleen and thymus, suggesting that the immunosuppression model is successfully established. However, CTX-induced inhibition is alleviated after administration of SCHPs-F1, and the spleen (4.44 ± 0.78 mg/g) and thymus (2.14 ± 0.60 mg/g) indices in the high-dose group are significantly higher than those in the model group (*p* < 0.01). The aforementioned information suggested that SCHPs-F1 encouraged the development of immunological organs in immunosuppressed mice.

### 2.3. Effects on Histological Changes of the Spleen and Thymus

**Figure 3:**
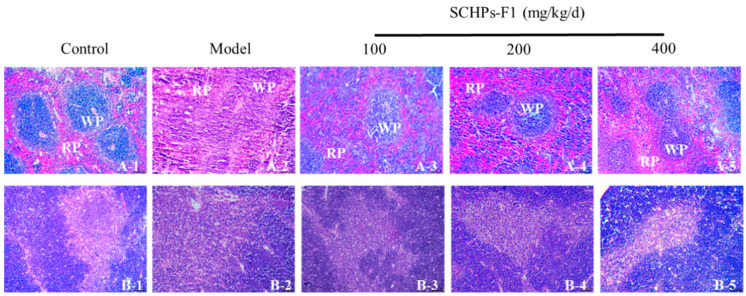
Effects of SCHPs-F1 on spleen (**A-1**–**A-5**) and thymus (**B-1**–**B-5**) histopathology (100× magnification). WP, white pulp; RP, red pulp.

The effect of SCHPs-F1 on immune organs is further verified by histomorphological observation (Figure 3). Staining findings reveal a distinct demarcation in the control group between splenic red and white pulp; the lymphocytes are neatly grouped, and the lymph nodes are manifest. In the model group, the germinal center of the spleen is obscure, the structure of white pulp is lowered, and the boundary region of white pulp and red pulp is blurring; the number of thymus lymphocytes is decreased, the cortex (dark areas) is shrunk, and the medulla (light areas) is increased. After SCHPs-F1 treatment, the injury of each group is alleviated. The histomorphological observation shows that SCHPs-F1 ameliorated the tissue injury and functional degradation of immune organs caused by CTX.

### 2.4. Effect of SCHPs-F1 on the Proliferation of Spleen Lymphocytes

**Figure 4:**
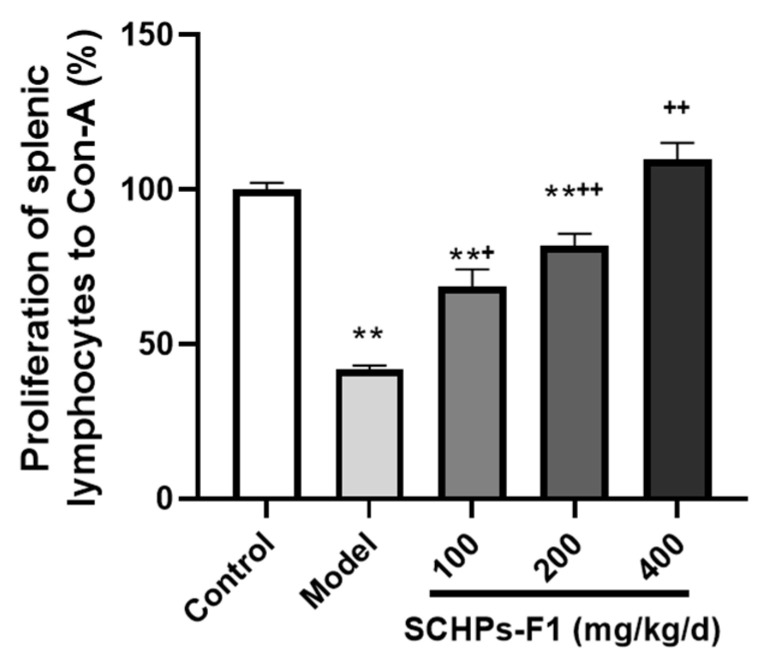
Effect of SCHPs-F1 on proliferation stimulation of spleen lymphocytes to Con-A in CTX-treated mice (*n* = 8). ** *p* < 0.01 vs. control group; ^+^ *p* < 0.05, ^++^ *p* < 0.01 vs. model group.

As shown in Figure 4, CTX dramatically inhibits the proliferative activity of mouse splenic lymphocytes. However, compared with the model group, SCHPs-F1 significantly enhances the induction impact of concanavalin A (Con-A), and the enhancement effect is most conspicuous in the high-dose group (*p* < 0.01), indicating that SCHPs-F1 could promote the proliferation activity of lymphocytes in the spleen of immunosuppressed mice.

### 2.5. Effect of SCHPs-F1 on NO Secretion in Peritoneal Macrophages

**Figure 5:**
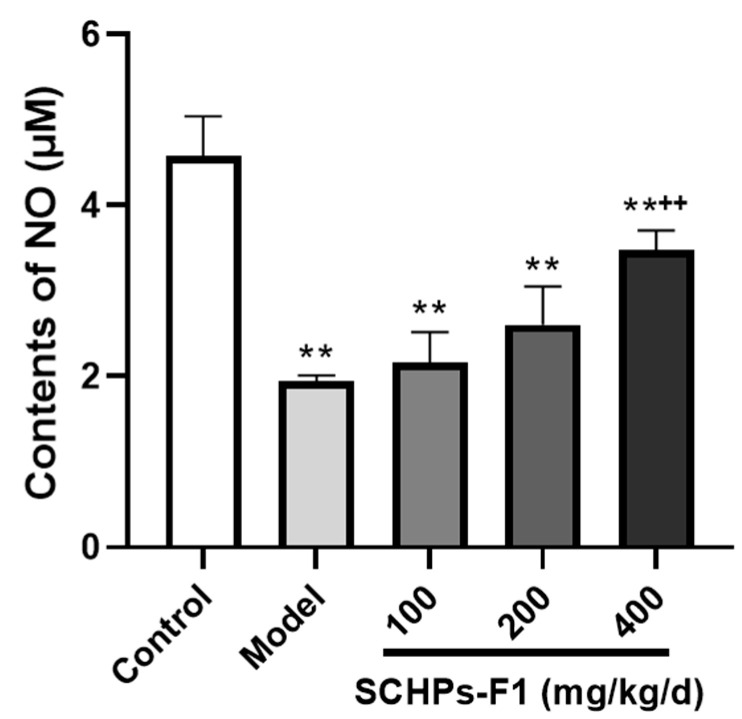
Effect of SCHPs-F1 on peritoneal microphages’ NO contents (*n* = 8). ** *p* < 0.01 vs. control group; ^++^ *p* < 0.01 vs. model group.

To further demonstrate the immunomodulatory effects of SCHPs-F1, the concentrations of NO in peritoneal macrophages were determined. In comparison with the control group, CTX treatment observably diminishes the content of NO in peritoneal macrophages (1.94 ± 0.07 vs. 4.58 ± 0.46, *p* < 0.01). However, SCHPs-F1 (100–400 mg/kg) reverses this situation in a concentration-dependent manner (Figure 5), and the NO level of peritoneal macrophages in the high-dose group is distinctly increased compared with the model group (3.48 ± 0.23 vs. 1.94 ± 0.07, *p* < 0.01).

### 2.6. Effects of SCHPs-F1 on Cytokine Production

**Figure 6:**
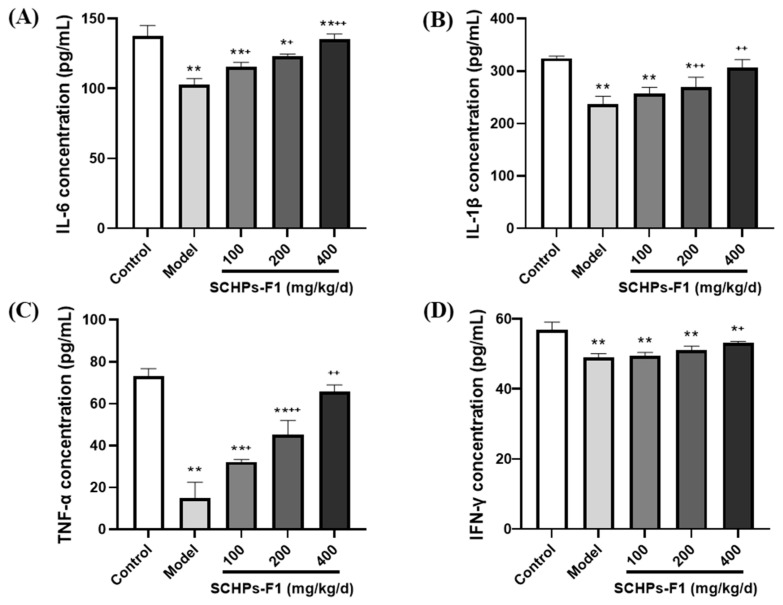
Effect of SCHPs-F1 on the levels of (**A**) IL-6, (**B**) IL-1β, (**C**) TNF-α, and (**D**) IFN-γ in CTX-treated mice (*n* = 8). * *p* < 0.05, ** *p* < 0.01 vs. control group; ^+^ *p* < 0.05, ^++^ *p* < 0.01 vs. model group.

Figure 6 demonstrates that the serum cytokine levels in the model group are markedly lower than those in the control group (*p* < 0.01), manifesting that CTX restrained the production of immunocompetent cytokines. Conversely, SCHPs-F1 can significantly augment the secretion of serum cytokines (IL-6, IL-1β, TNF-α, and IFN-γ) compared with the CTX group. At the dosage of 400 mg/kg SCHPs-F1, these cytokine levels are obviously increased, in which IL-6 increased by 31.71% (from 102.50 to 135.00 pg/mL), IL-1β increased by 29.34% (from 236.69 to 306.14 pg/mL), TNF-α increased by 345.67% (from 14.78 to 65.87 pg/mL), and IFN-γ increased by 8.68% (from 48.94 to 53.19 pg/mL).

### 2.7. Effects of SCHPs-F1 on Humoral Immunity

**Figure 7:**
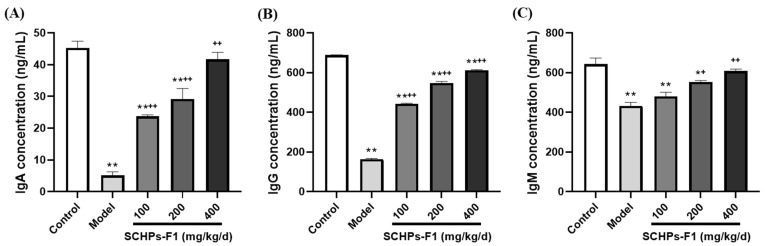
Effect of SCHPs-F1 on the levels of (**A**) IgA, (**B**) IgG, and (**C**) IgM in CTX-treated mice (*n* = 8). * *p* < 0.05, ** *p* < 0.01 vs. control group; ^+^ *p* < 0.05, ^++^ *p* < 0.01 vs. model group.

According to Figure 7, the activities of three immunoglobulins are manifestly suppressed under the influence of CTX compared with the control group (5.17 ± 1.07 vs. 45.29 ± 2.14 ng/mL, IgA; 163.62 ± 3.81 vs. 688.64 ± 1.22 ng/mL, IgG; 432.2 ± 18.13 vs. 643.81 ± 29.91 ng/mL, IgM; *p* < 0.01). After 400 mg/kg SCHPs-F1 treatment, the serum immunoglobulin concentration is prominently enhanced compared with the CTX group (*p* < 0.01), in which IgA increased by 706.38% (from 5.17 to 41.69 ng/mL), IgG increased by 273.67% (from 163.62 to 611.40 ng/mL), and IgM increased by 40.70% (from 432.20 to 608.10 ng/mL). These results suggest that SCHPs-F1 can counteract CTX-induced immunosuppression by restoring immunoglobulin levels.

### 2.8. Effects of SCHPs-F1 on the NF-κB and MAPK Pathway in the Spleen

**Figure 8:**
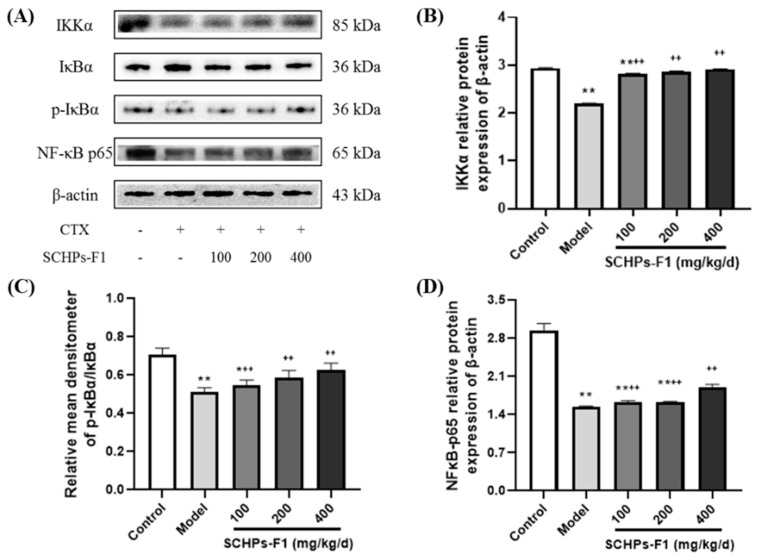
Effects of SCHPs-F1 on the protein expression levels in the NF-κB pathway in spleens of CTX-treated mice. (**A**) The result of western blot; (**B**) IKKα relative protein expression of β-actin; (**C**) Relative mean densitometer of p-IκBα/IκBα; (**D**) NF-κB p65 relative protein expression of β-actin. * *p* < 0.05, ** *p* < 0.01 vs. control group; ^++^ *p* < 0.01 vs. model group.

The results show that the expression levels of IKKα and NF-κB p65 in the CTX treatment group are considerably lower than in the control group, and the degree of phosphorylation levels of IκBα is also attenuated (Figure 8, *p* < 0.01). After treatment with SCHPs-F1 (100–400 mg/kg), the protein expression levels of IKKα, p-IκBα, and NF-κB p65 in spleen tissues are noticeably enhanced, and the degradation of IκBα protein is augmented, which is a substantial difference in comparison with the CTX group (*p* < 0.01). These results suggest that SCHPs-F1 can regulate the protein content in the NF-κB pathway, thereby alleviating the immunosuppression induced by CTX in mice.

**Figure 9:**
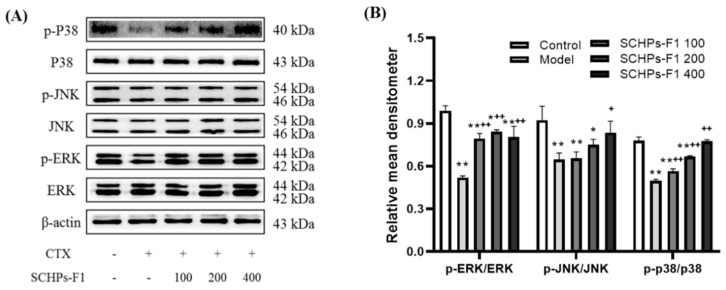
Effect of SCHPs-F1 on the protein expression levels of MAPK pathway in spleens of CTX-treated mice. (**A**) The result of western blot; (**B**) Relative mean densitometer of p-ERK/ERK, p-JNK/JNK, p-p38 p38. * *p* < 0.05, ** *p* < 0.01 vs. control group; ^+^ *p* < 0.05, ^++^ *p* < 0.01 vs. model group.

To further validate the immunomodulatory mechanism of SCHPs-F1, the degree of protein phosphorylation in the MAPK pathway was detected. Compared with the control, CTX treatment significantly down-regulates the phosphorylation levels of JNK, ERK, and p38 (Figure 9). However, the phosphorylation levels of JNK, ERK, and p38 are distinctly elevated under the influence of 400 mg/kg SCHPs-F1 compared with the model group (*p* < 0.05). Thus, SCHPs-F1 could activate the immunomodulatory ability of the mouse spleen by activating the MAPK pathway.

## 3. Discussion

The immune system is a defense network surrounding the whole body, which is responsible for resisting the invasion of harmful substances and maintaining the health of the body [1]. Effective immune regulation can keep the immune response at the optimal level to maintain dynamic physiological balance and internal environment stability [24]. Immunomodulators restore the originally disordered immune response to a normal level by regulating the immune function of the body, and immunotherapy has gradually become the first-line treatment for cancer [25,26]. At present, the exploitation of potential immunomodulatory peptides from a range of dietary resources has sparked great interest [27]. Low molecular weight peptides have been proven to exert immunomodulatory effects such as boosting macrophage proliferation, cytokine production, and inflammatory mediators in a growing number of investigations [28,29]. Therefore, in this study, we investigated the immunomodulatory effect of the SCHPs-F1 (less than 1 kDa) extracted from the head of *S. crassicornis* on CTX-induced immunosuppressed mice.

As a clinical antitumor drug, CTX has a strong immunosuppressive effect [30]. However, long-term usage of CTX might produce untoward effects, such as bone marrow suppression and cardiotoxicity [31]. So, CTX was often used to establish immunosuppressive mouse models [17]. According to our results, CTX could decrease the murine spleen and thymus index and reduce NO, serum cytokines, and immunoglobulins levels, successfully demonstrating the feasibility of the CTX-induced immunosuppression model.

The immune organ is the primary location of the immune response, which carries out defensive reactions to ensure that noxious stimuli are removed. The thymus and spleen are the sites of immune cell differentiation, maturation, and immune response as two important immune organs in the human body [32]. In this study, SCHPs-F1 treatment reversed CTX-induced weight loss and immune organ atrophy. In addition, after SCHPs-F1 treatment, the morphology of the thymus gland in each group was improved, the number of mature lymphocytes in the cortical area was increased, and the spleen injury was also recovered. These results indicated that SCHPs-F1 has an obvious repairing effect on immune organs.

Activation and proliferation of lymphocytes are essential components of the immune response [33]. As another indispensable immune cell, macrophages play an irreplaceable physiological and pathological role in all endocrine tissues of the body [34]. NO has become an important intracellular and intercellular regulatory molecule in the cytotoxicity or inhibition of macrophages to target cells [35]. Moreover, NO is also involved in T cell-mediated immune processes [36]. In our present studies, CTX caused rapid clearance of lymphocytes, which was similar to previous studies [37]. However, SCHPs-F1 significantly promoted the proliferation of T lymphocytes, indicating that SCHPs-F1 could enhance cellular immunity in immunosuppressed mice. In addition, SCHPs-F1 (400 mg/kg) treatment significantly increased the secretion of NO by macrophages. These results suggested that SCHPs-F1 could protect immune cells from CTX damage and ameliorates their viability.

Cytokines play a variety of biological functions as signal transduction substances, and the regulation of cytokine content is essential to maintain the homeostasis of the immune system [34]. IL-6 can exhibit a pro-inflammatory effect by augmenting the release of IL-1β, and IFN-γ can increase the activity of TNF-α and promote the synthesis of NO [38]. Moreover, IL-1β and TNF-α also play key roles in cell apoptosis, cell proliferation, and immune response [39]. In this study, SCHPs-F1 treatment (400 mg/kg) significantly increased the content of serum cytokines in immunosuppressed mice (*p* < 0.01), indicating that SCHPs-F1 could strengthen the immunoreaction by upregulating the secretion of serum cytokines. Immunoglobulins are a kind of immune active molecules involved in humoral immunity, which can combine with antigens to form complexes and block the harm of pathogens to the body [40]. Our results demonstrated that SCHPs-F1 treatment (400 mg/kg) remarkably increased the serum immunoglobulin levels, suggesting that SCHPs-F1 might exert an immunomodulatory effect on CTX-treated mice through humoral immunity.

**Figure 10:**
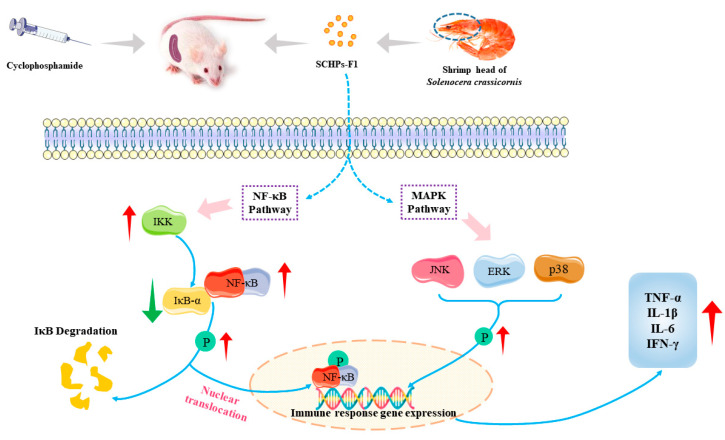
SCHPs-F1 ameliorates CTX-induced immunosuppression, possibly by regulating the NF-κB and MAPK pathways.

It has been reported that bioactive peptides can interfere with NF-κB and MAPK signaling pathways to a certain extent, which provides new ideas for immunotherapy drugs [41,42]. NF-κB is an inducible nuclear transcription factor that not only modulates the expression of diverse inflammatory mediators in innate immune cells but also mediates the proliferation and activation of T lymphocytes [43], which is important in regulating both immune and inflammatory responses. MAPK is an important intermediary in the activation of cytokines and neurotransmitters, assisting in the transmission of signals from the cell surface to the interior of the nucleus, thereby participating in immune regulation [44]. Our results showed that SCHPs-F1 could activate the NF-κB signaling pathway and enhance the protein expression levels. In addition, SCHPs-F1 also elevated the phosphorylation levels of proteins (JNK, ERK, and p38) in the MAPK pathway. These results suggested that SCHPs-F1 might perform immunomodulatory functions via activating the NF-κB and MAPK pathways, thereby ameliorating the CTX-induced immunosuppression (Figure 10).

Studies have shown that the immunomodulatory properties of bioactive peptides depend on the modulation of cytokines, the production of antibodies, and the stimulation of reactive oxygen species in the immune system [45]. In addition, the content of characteristic amino acids, peptide sequence, length, and hydrophobicity are also closely related to immunomodulatory activity [27,46]. Among them, low molecular weight protein hydrolyzates and peptides containing a large number of hydrophobic amino acids have been shown to boost immune regulation [47]. Furthermore, branched-chain amino acids (leucine, isoleucine, and valine) have been proven to activate the mTOR signaling pathway, which is engaged in the enhancement of both innate and adaptive immunological responses [48], and the hydrophobic structure of branched-chain amino acids also conforms to the characteristics of immunomodulatory peptides. Therefore, it was crucial to determine the immunomodulatory peptide sequence in SCHPs-F1 in accordance with the structural characteristics of the immunomodulatory peptide so as to further prove the immunomodulatory function of SCHPs-F1. In our study, the SCHPs-F1 contained a large number of branched-chain amino acids (leucine: 5.90 g/100 g; valine: 4.10 g/100 g; isoleucine: 3.94 g/100 g), which might be another reason contributing to the immune enhancement of SCHPs-F1. However, a clear link between low molecular weight peptides in SCHPs-F1 and immunomodulatory effects needs to be further analyzed through techniques such as molecular docking. The immunomodulatory mechanism of the target peptides also needs to be further investigated in vitro and in vivo.

## 4. Materials and Methods

### 4.1. Materials and Reagents

*S. crassicornis* was provided by the Zhoushan International Aquatic Center (Zhoushan, China). CTX was purchased from Aladdin Biochemical Technology Co., Ltd. (Shanghai, China). Hematoxylin and Eosin (H&E) staining kit and Con A were supplied from Beyotime Biotechnology (Shanghai, China). Rabbit antibody NF-κB p65, IKKα, IκBα, phospho-IκBα, phospho-NF-κB p65, JNK phospho-JNK, ERK, phospho-ERK, p38, and phospho-p38 were supplied by Cell Signaling Technology (Boston, MA, USA); β-actin were supplied by Solarbio (Beijing, China).

### 4.2. Animal Treatment

**Figure 11:**
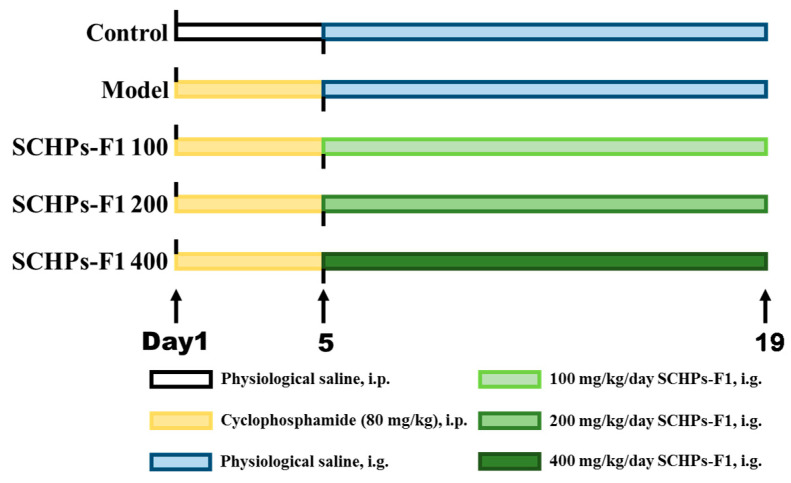
The experimental program and treatment of mice group. i.p., intraperitoneal injection; i.g., intragastric administration.

A total of 40 ICR mice (six-weeks-old, 20 ± 2 g) were purchased from the Zhejiang Province Laboratory Animal Public Service Platform (Hangzhou, China). The protocol for the care and use of experimental animals was approved by the Animal Ethics Committee of Zhejiang Ocean University. All mice were kept under standard feeding conditions (humidity: 60 ± 5%; temperature: 22 ± 2 °C). After 7 days of adaptation, mice were randomly separated into five groups (*n* = 8). The control group was given normal saline for 19 consecutive days. The remaining groups were intraperitoneally injected with CTX (80 mg/kg/d) for 5 days, and then given normal saline or diverse dosages of SCHPs-F1 (100 mg/kg/d, 200 mg/kg/d, and 400 mg/kg/d) for the following 14 days (Figure 11).

### 4.3. Analysis of Body Weight and Immune Organ Index

The daily weight changes of mice were recorded. Thymus and spleen tissues were removed and immediately weighed after the mice were executed by cervical dislocation. The following formula is used to compute the immune organ index: thymus or spleen index (mg/g) = thymus or spleen weight (mg)/body weight (g).

### 4.4. Histomorphology

Partial thymus and spleen tissues were fixed with paraformaldehyde (4%) and embedded in paraffin. Organ specimens were cut into 4 μm slices, and H&E staining was performed as described in the preceding research [49,50]. The staining status of sections was observed by a Biological Microscope CX31 (Olympus, Tokyo, Japan).

### 4.5. Splenic Lymphocyte Proliferation Assay

Splenic lymphocyte suspensions were prepared under aseptic conditions, and cell proliferation rates were determined with reference to the approach described by Ren and colleagues [51]. Briefly, the spleen was chopped, ground, and sieved through a stainless steel mesh (200 mesh). Then, the cell screen was rinsed with RMPI-1640 medium (without serum), and the supernatant was removed after centrifugation of the suspension (1500 rpm, 15 min). Red blood cell (RBC) lysis buffer was added to the reaction for 5 min, then RMPI-1640 medium (without serum) was added, and the supernatant was discarded after centrifugation (1500 rpm, 15 min). Subsequently, the cells were resuspended in RMPI-1640 medium (containing 10% fetal bovine serum) and incubated for 4 h to collect suspended and non-adherent cells. Spleen lymphocytes of mice in each group were sorted into the control group and Con A (5 μg/mL) treatment group, then placed in 96-well plates (5 × 10^5^ cells/mL) with three repeated wells in each group and cultured for 24 h at 37 °C in an incubator (Forma 3111 CO_2_ incubator, Thermo Forma, Waltham, MA, USA) containing 5% CO_2_. Then, 200 μL MTT staining solution was added to the culture for 4 h in the dark. Subsequently, the MTT reagent was removed, and 150 μL DMSO was added to oscillate for 15 min. Finally, the absorbance at 490 nm of each well was determined by a SpectraMax M2 microplate reader (Molecular Devices, Silicon Valley, CA, USA).

### 4.6. Measurement of NO Production

The NO synthesis capacity of mouse peritoneal macrophages was detected using the protocol followed in previous studies [52]. Peritoneal cells of mice were collected under aseptic circumstances and resuspended in a medium containing 10% fetal bovine serum, then incubated at 37 °C with 5% CO_2_ for 4 h. Subsequently, adherent cells were collected, and the cells with adjusted density were inoculated into 96-well plates (2 × 10^5^ cells/mL). 50 μL of upper culture medium was absorbed after 24 h, equal volume Griess reagents were successively added, and the absorbance of each group was recorded at 540 nm.

### 4.7. Assay of Cytokines and Immunoglobulins in Serum

Blood samples were taken from the mouse orbit and centrifuged (6000× *g*, 5 min) to acquire serum. The concentrations of cytokines and immunoglobulins in the serum were examined by ELISA kits in accordance with the manufacturer’s directions (Boster, Wuhan, China).

### 4.8. Western Blotting

The milled spleen tissue was treated with cell lysis solution supplemented with a protease inhibitor and homogenized in ice-cold PBS. Then, the supernatant was collected after centrifugation (10,000× *g*, 4 °C, 10 min). The BCA protein assay kit was used to detect protein content, and the Western blot was performed with reference to the method of the prior investigation [53,54]. The gel separated by electrophoresis was transferred to the PVDF membrane, then 5% defatted milk powder was added to seal the membrane for 1 h. The membrane was incubated with the corresponding primary and secondary antibodies, respectively. Then, target imprints were detected using the enhanced chemiluminescent (ECL) kit (Boster, Wuhan, China).

### 4.9. Statistical Analysis

All experimental data were analyzed with SPSS 24.0 software and represented as mean ± standard deviation (x ± s). ANOVA one-way analysis of variance was used to examine the data for statistical significance. The *p*-values < 0.05 indicates that the difference between the means is significant.

## 5. Conclusions

In summary, the current investigation demonstrated that SCHPs-F1 could ameliorate CTX-induced immunosuppression. SCHPs-F1 could recover the immune organ index and histopathological changes of the spleen, stimulating the proliferation of splenic lymphocytes and the secretion of NO by macrophages, increasing the serum cytokines (IL-6, IL-1β, TNF-α, and IFN-γ) and immunoglobulin (IgA, IgG and IgM) levels. Meanwhile, the immunomodulatory mechanism of SCHPs-F1 was probably linked to the activation of the NF-κB and MAPK pathways. In addition, SCHPs-F1 contained a large number of branched-chain amino acids, which may be another important reason for its immunomodulatory function. Further studies are needed to screen peptides with immunomodulatory effects in terms of molecular docking, and the immunomodulatory mechanism of the screen peptides will be investigated in vitro and in vivo in the future.

## Figures and Tables

**Table 1:** Effect of SCHPS-F1 on immune organ indices in CTX-induced mice (*n* = 8).

Group	The Spleen Index (mg/g)	The Thymus Index (mg/g)
Control	4.48 ± 1.55	2.35 ± 0.80
Model	2.79 ± 1.14 *	1.86 ± 0.53 **
SCHPs-F1 100	3.78 ± 0.50	1.93 ± 0.28 *
SCHPs-F1 200	4.14 ± 1.37 ^+^	2.04 ± 0.42 ^+^
SCHPs-F1 400	4.44 ± 0.78 ^++^	2.14 ± 0.60 ^++^

* *p* < 0.05, ** *p* < 0.01 vs. control group; ^+^ *p* < 0.05, ^++^ *p* < 0.01 vs. model group.

## Data Availability

Data supporting our findings can be sent upon request.

## Supplementary Materials

The following supporting information can be downloaded at: https://www.mdpi.com/article/10.3390/ijms241210297/s1.
