# Supplementary material for: Effects of Low Molecular Weight Peptides from Red Shrimp (Solenocera crassicornis) Head on Immune Response in Immunosuppressed Mice
Source: Int J Mol Sci. 2023 Jun 18;24(12):10297. doi: 10.3390/ijms241210297 (PMC10299419; doi:10.3390/ijms241210297)
Supplement: Supplementary file 1 [file ijms-24-10297-s001.zip › ijms-2434376-supplementary.docx]

Supplementary data

**Table S1.** A total of 71 peptides were identified from SCHPs-F1 by LC-MS/MS.

| **Retention Time (min)** | **Sequence** | **Length** | **Charges** | **m/z** | **Calculated Mass** | **Exact Mass** |
| --- | --- | --- | --- | --- | --- | --- |
| 0.58 | LR | 2 | 1 | 288.20302 | 287.36 | 287.19574 |
| 0.60 | AQ | 2 | 1 | 218.11353 | 217.22 | 217.10626 |
| 0.60 | AR | 2 | 1 | 246.15607 | 245.27 | 245.14879 |
| 0.60 | LK | 2 | 1 | 260.19687 | 259.35 | 259.18959 |
| 0.61 | LH | 2 | 1 | 269.16082 | 268.32 | 268.15354 |
| 0.62 | MS | 2 | 1 | 237.09035 | 236.28 | 236.08308 |
| 0.65 | ET | 2 | 1 | 249.10811 | 248.23 | 248.10084 |
| 0.66 | TH | 2 | 1 | 257.12443 | 256.26 | 256.11716 |
| 0.67 | TK | 2 | 1 | 248.16048 | 247.29 | 247.15321 |
| 0.78 | LGINS | 5 | 2 | 252.14483 | 502.56 | 502.27511 |
| 0.89 | NS | 2 | 1 | 220.0928 | 219.19 | 219.08552 |
| 0.89 | TQTL | 4 | 2 | 231.63156 | 461.51 | 461.24856 |
| 0.95 | VH | 2 | 1 | 255.14517 | 254.29 | 254.13789 |
| 0.99 | MH | 2 | 1 | 287.11724 | 286.35 | 286.10996 |
| 1.11 | VGTAL | 5 | 2 | 230.64193 | 459.53 | 459.2693 |
| 1.13 | RE | 2 | 1 | 304.16155 | 303.31 | 303.15427 |
| 1.14 | LSN | 3 | 1 | 333.17686 | 332.35 | 332.16958 |
| 1.16 | FS | 2 | 1 | 253.11828 | 252.26 | 252.11101 |
| 1.16 | PH | 2 | 1 | 253.12952 | 252.27 | 252.12224 |
| 1.28 | AK | 2 | 1 | 218.14992 | 217.26 | 217.14264 |
| 1.39 | EY | 2 | 1 | 311.12376 | 310.20 | 310.11649 |
| 1.52 | LE | 2 | 1 | 261.1445 | 260.29 | 260.13722 |
| 1.54 | TV | 2 | 1 | 219.13393 | 218.25 | 218.12666 |
| 1.56 | SY | 2 | 1 | 269.1132 | 268.16 | 268.10592 |
| 1.84 | LSNT | 4 | 1 | 435.20856 | 433.45 | 434.20128 |
| 1.86 | AL | 2 | 1 | 203.13902 | 202.25 | 202.13174 |
| 1.97 | FQ | 2 | 1 | 294.14483 | 293.32 | 293.13756 |
| 2.16 | EL | 2 | 1 | 261.1445 | 260.29 | 260.13722 |
| 2.16 | KL | 2 | 1 | 260.19687 | 259.35 | 259.18959 |
| 2.29 | VM | 2 | 1 | 249.12674 | 248.34 | 248.11946 |
| 2.32 | NL | 2 | 1 | 246.14483 | 245.28 | 245.13756 |
| 2.46 | GL | 2 | 1 | 189.12337 | 188.23 | 188.11609 |
| 2.62 | VY | 2 | 1 | 281.14958 | 280.22 | 280.14231 |
| 2.64 | ISE | 3 | 1 | 348.17653 | 347.36 | 347.16925 |
| 2.66 | WS | 2 | 1 | 292.12918 | 291.30 | 291.12191 |
| 3.31 | AF | 2 | 1 | 237.12337 | 236.26 | 236.11609 |
| 3.35 | MP | 2 | 1 | 247.11109 | 246.32 | 246.10381 |
| 3.38 | TL | 2 | 1 | 233.14958 | 232.28 | 232.14231 |
| 3.40 | GF | 2 | 1 | 223.10772 | 222.24 | 222.10044 |
| 3.59 | SF | 2 | 1 | 253.11828 | 252.26 | 252.11101 |
| 3.63 | DF | 2 | 1 | 281.1132 | 280.27 | 280.10592 |
| 3.82 | VF | 2 | 1 | 265.15467 | 264.32 | 264.14739 |
| 3.85 | TF | 2 | 1 | 267.13393 | 266.29 | 266.12666 |
| 4.03 | WNQ | 3 | 1 | 448.18267 | 446.46 | 447.1754 |
| 4.29 | YY | 2 | 1 | 345.1445 | 344.16 | 344.13722 |
| 4.59 | LVT | 3 | 1 | 332.218 | 331.41 | 331.21072 |
| 4.93 | GW | 2 | 1 | 262.11862 | 261.28 | 261.11134 |
| 4.97 | FGT | 3 | 1 | 324.1554 | 323.34 | 323.14812 |
| 5.03 | SW | 2 | 1 | 292.12918 | 291.30 | 291.12191 |
| 5.48 | EW | 2 | 1 | 334.13975 | 333.34 | 333.13247 |
| 5.48 | QW | 2 | 1 | 333.15573 | 332.36 | 332.14846 |
| 5.52 | TW | 2 | 1 | 306.14483 | 305.33 | 305.13756 |
| 5.58 | SNETFKFSS | 9 | 2 | 523.7431 | 1046.05 | 1045.4716 |
| 5.71 | WP | 2 | 1 | 302.14992 | 301.34 | 301.14264 |
| 6.07 | LY | 2 | 1 | 295.16523 | 294.25 | 294.15796 |
| 7.17 | FY | 2 | 1 | 329.14958 | 328.26 | 328.14231 |
| 7.27 | LLER | 4 | 2 | 265.66847 | 529.63 | 529.3224 |
| 8.25 | YF | 2 | 1 | 329.14958 | 328.26 | 328.14231 |
| 9.35 | LGGNL | 5 | 1 | 474.25584 | 472.54 | 473.24856 |
| 9.35 | LVM | 3 | 1 | 362.2108 | 361.50 | 361.20353 |
| 9.50 | LGAV | 4 | 1 | 359.2289 | 358.43 | 358.22162 |
| 9.94 | LW | 2 | 1 | 318.18122 | 317.39 | 317.17394 |
| 10.24 | LGGL | 4 | 1 | 359.2289 | 358.44 | 358.22162 |
| 10.80 | LSNTL | 5 | 1 | 548.29262 | 546.61 | 547.28534 |
| 11.08 | LLILP | 5 | 1 | 568.40686 | 567.77 | 567.39958 |
| 11.10 | DLPAP | 5 | 1 | 512.27149 | 511.55 | 511.26421 |
| 11.12 | WL | 2 | 1 | 318.18122 | 317.39 | 317.17394 |
| 11.26 | LL | 2 | 1 | 245.18597 | 244.34 | 244.17869 |
| 11.83 | FW | 2 | 1 | 352.16557 | 351.40 | 351.15829 |
| 12.23 | LDW | 3 | 1 | 433.20816 | 432.47 | 432.20088 |
| 12.58 | VLF | 3 | 1 | 378.23873 | 377.48 | 377.23146 |
